# Genomic analyses reveal high diversity and rapid evolution of *Pichia kudriavzevii* within a neonatal intensive care unit in Delhi, India

**DOI:** 10.1128/aac.01709-24

**Published:** 2025-01-24

**Authors:** Kusum Jain, Yue Wang, Peeyush Jain, Barsha Kalita, Raju Shivarathri, Manju Chauhan, Hardeep Kaur, Neeraj Chauhan, Jianping Xu, Anuradha Chowdhary

**Affiliations:** 1 Medical Mycology Unit, Department of Microbiology, Vallabhbhai Patel Chest Institute, University of Delhi 28742 , Delhi, India; 2 Department of Zoology, Ramjas College, University of Delhi 28742 , Delhi, India; 3 National Reference Laboratory for Antimicrobial Resistance in Fungal Pathogens, Vallabhbhai Patel Chest Institute, University of Delhi 28742 , Delhi, India; 4 Department of Biology, McMaster University 98616 , Hamilton, Ontario, Canada; 5 Department of Paediatrics, Hindu Rao Hospital and NDMC Medical College 56888 , Delhi, India; 6 Center for Discovery and Innovation, Hackensack Meridian Health, Nutley, New Jersey, USA; University of Iowa, Iowa City, Iowa, USA

**Keywords:** *Pichia kudriavzevii*, neonate, loss of heterozygosity, phenotypic divergence, transcriptome, fluconazole resistance, itraconazole resistance, ploidy

## Abstract

*Pichia kudriavzevii* causes life-threatening infections in immunocompromised hosts, including hospitalized neonates. This pathogen is intrinsically resistant to fluconazole, while uncommon *P. kudriavzevii* strains resistant to multiple antifungal drugs, including voriconazole, amphotericin B, and echinocandins, have also been reported from healthcare environments. Thus, understanding how *P. kudriavzevii* spread, persist, and adapt to healthcare settings could help us develop better infection management strategies. In this study, whole genome sequencing identifies multiple outbreaks of bloodstream infections in a single neonatal intensive care unit (NICU) over 5 years caused by genetically diverse strains of *P. kudriavzevii*. Interestingly, two genetically distinct clusters of *P. kudriavzevii* strains showed frequent loss of heterozygosity (LOH) events between two temporal samples. The first outbreak cluster (2015–2016) showed LOH at chromosomes 1, 4, and 5, and the other outbreak cluster (2020) exhibited LOH at chromosome 2. The circulation of two separate strain clusters of *P. kudriavzevii* suggests nosocomial transmission in the NICU in different time periods. Furthermore, we compared the transcriptomic profiles of three isolates of clusters I and II that exhibited distinct fluconazole and itraconazole MICs. While no significant difference in gene expression was found at the azole-target gene *ERG11* or the ATP-binding cassette (ABC) transporter genes, such differences were found in genes involved in cell division and filamentation, such as *SIR2* (sirtuin deacetylase) and *RFA1* (replication factor A). Interestingly, increased filamentation was observed in clade I isolate exhibiting high fluconazole MICs. Together, our study indicates significant diversity, persistence, and rapid evolution of *P. kudriavzevii* within a single NICU.

## INTRODUCTION

Fungal bloodstream infections (BSIs) are a major group of healthcare-associated infections, with causal agents increasingly shifting to non-*albicans Candida* (NAC) species (1, 2). Among the NACs, *Pichia kudriavzevii* (formerly *Candida krusei*) is intrinsically resistant to fluconazole (FLU), a first-line antifungal agent commonly used in low resource settings for treating candidemia (3, 4). In addition, *P. kudriavzevii* is among the most common species causing BSIs in patients with hematologic malignancies in North America and Europe (5, 6). Importantly, patients with both hematologic malignancies and BSIs by *P. kudriavzevii* have a very high-crude 12-week mortality (52.9%) (7). Aside from being intrinsically resistant to FLU, strains of *P. kudriavzevii* are frequently reported as resistant to voriconazole (VRC) and echinocandins, severely limiting therapeutic choices for treating such infections. For example, during treatments, acquired resistance to VRC was reported in a renal transplant patient with urinary tract infection due to *P. kudriavzevii*, and acquired resistance to echinocandins was reported in patients with acute myelogenous leukemia, highlighting the need to monitor *P. kudriavzevii* infections during antifungal treatments for their potential development of resistance (5, 8, 9). A multicenter study including 10 university hospitals in Iran reported that 33.3% and 13.3% clinical isolates of *P. kudriavzevii* during the period of 2019–2021 were multi-azole (FLU, itraconazole, and posaconazole)- and multidrug (caspofungin and VRC)-resistant, respectively (10). Another cross-sectional study from Cameroon reported that 32.5% of *P. kudriavzevii* isolates from HIV-infected patients were resistant to azole, 5-flucytosine (5-FC), and amphotericin B (AMB) drugs (11). Together, these studies indicate that the emergence of less common yet potentially multidrug-resistant (MDR) strains of *P. kudriavzevii* is a serious concern. Understanding how they spread, persist, and adapt to healthcare settings could help us develop better management strategies.

In the last decade, *P. kudriavzevii* has caused multiple outbreaks of BSIs in neonatal intensive care units (NICUs) in low- and middle-income countries, such as Brazil, India, and South Africa (12–16). In India, *P. kudriavzevii* ranks as the sixth most common cause of BSIs in adult intensive care units (ICUs) (17). Additionally, sporadic outbreaks of nosocomial BSIs in the NICUs are widely reported from India (13, 14, 16). However, the genetic population of *P. kudriavzevii* causing such outbreaks remains largely unknown. In the present study, we used whole genome sequencing (WGS) to examine the genetic structure of a *P. kudriavzevii* population causing BSIs spanning a period of 5 years (2015–2020) in a single NICU in Delhi, India. Furthermore, to determine the potential mechanism of antifungal resistance, a comparative transcriptomic analysis was performed from isolates belonging to two distinct genetic clusters exhibiting different minimum inhibitory concentrations (MICs) against FLU.

## RESULTS

### Isolates

A total of 165 clinical isolates of *P. kudriavzevii* obtained from 16 tertiary care hospitals located in Delhi and adjoining National Capital Region (NCR) were collected in an ongoing antifungal resistance surveillance during a period of 8 years from 2015 to 2023. *Pichia kudriavzevii* represents the fifth most common agent of BSIs in our ongoing antifungal surveillance in Delhi and adjoining NCR (unpublished data) during the last 8 years. A major proportion (80%) of isolates was obtained from cases of BSIs (*n* = 132). Importantly, 57% of BSI cases (*n* = 75) occurred in the NICUs of two hospitals, and the remaining cases of BSIs were reported from adult medical ICUs of 14 hospitals. Among the *P. kudriavzevii* isolates obtained from two NICUs, we investigated the genomic epidemiology of a protracted outbreak involving 24 hospital-born neonates between 2015 and 2020 in a 24-bed capacity NICU within a single multispecialty hospital in Delhi, India.

### *In vitro* susceptibility profile of *P. kudriavzevii* isolates (*n* = 165)

Overall, the high geometric mean (GM) MIC value 17.7 mg/L of FLU was observed within the total population of 165 strains (Table 1). Among the 165 isolates, 144 (87.3%) exhibited high MIC values (MIC range of 16–32 mg/L), and 21 (12.7%) had low MIC values for FLU (4–8 mg/L). Interestingly, out of these 21 isolates, 16 showed a FLU MIC value of 8 mg/L, whereas five showed 4 mg/L FLU MIC. AFST for FLU was repeated 10 times for these 21 isolates, and the median MIC value was calculated. The MIC data of three isolates that originally had MIC of 4 mg/L increased to 8 mg/L. Together, the additional test revealed that 19 isolates had median MIC value of 8 mg/L, and only two displayed median MIC values of 4 mg/L. Overall, the MIC distribution of 165 isolates for FLU showed a Gaussian (normal) pattern with 98.7% isolates exhibiting MICs either one well below or above the mean MIC value (16 mg/L). Of note, 8.2% of isolates (*n* = 14) were found to be non-wild type for itraconazole (ITC, MIC ≥ 1 mg/L). Among the other azoles tested, isavuconazole (ISA) showed the highest potency against *P. kudriavzevii* isolates with GM-MICs 0.06 mg/L, followed by VRC (GM-MIC 0.06 mg/L). In the case of two echinocandins, anidulafungin (AFG; GM-MIC 0.09 mg/L) was more potent than the micafungin. The modal MICs of AMB were 0.5 mg/L.

**TABLE 1:** *In vitro* antifungal susceptibility profile of 165 *Pichia kudriavzevii* isolates against nine antifungal drugs by using broth microdilution method (CLSI M27-A3)

Drug^*a*^	MIC (mg/L)													Range	GM^*b*^	MIC_50_^*c*^	MIC_90_^*d*^
	0.01	0.03	0.06	0.12	0.25	0.5	1	2	4	8	16	32	64				
FLU									2	19	87	57		4–32	17.50	16	32
ITC	1	16	15	42	34	43	11	3						0.01–2	0.20	0.25	0.5
VRC		27	49	59	20	10								0.03–0.5	0.09	0.12	0.25
ISA	14	31	51	54	12	3								0.01–0.5	0.06	0.06	0.12
POS	3	4	9	30	77	42								0.01–0.5	0.21	0.25	0.5
AMB			5	35	24	46	55							0.06–1	0.39	0.5	1
MFG	5	3	35	81	28	13								0.01–0.5	0.12	0.12	0.25
AFG	6	25	59	47	12	16								0.01–0.5	0.08	0.06	0.25
5-FC			1			1		15	33	73	14			0.06–16	5.69	8	8

^*a*^ FLU, fluconazole ITC, itraconazole; VRC, voriconazole; ISA, isavuconazole; POS, posaconazole; AMB, amphotericin B; MFG, micafungin; AFG, anidulafungin; 5 FC, flucytosine.

^*b*^ Geometric mean of minimum inhibitory concentrations.

^*c*^ MIC_50_, minimum inhibitory concentrations against 50% of strains.

^*d*^ MIC_90_, minimum inhibitory concentrations against 90% of strains.

### *Pichia kudriavzevii* in NICU

In the 2015–2020 five-year span, a total of 244 neonatal blood culture samples were collected from this NICU, of which 67 (27.4%) had BSIs. The first case of BSI due to *P. kudriavzevii* was observed in 2015, and three cases were reported in 2016 (*n* = 3), followed by a large increase in 2018–19 (*n* = 11) and a clustering of 10 cases in 2020. During 2015–2020, at this NICU, *P. kudriavzevii* was the most common (*n* = 25) fungal species cultured from blood specimens, followed by *Candida tropicalis* (*n* = 21), *Candida albicans* (*n* = 9), *Candida blankii* (*n* = 9), *Nakaseomyces glabrata* (*n* = 1), *Candida parapsilosis* (*n* = 1), and *Rhodotorula mucilaginosa* (*n* = 1). The mean frequency of *P. kudriavzevii* BSI cases over the 5 years was 4.8 per year with 0 case in 2017 and relatively few cases of *P. kudriavzevii* (*n* = 4) in 2015–2016. However, from 2018 to 2020, cases of *C. blankii* decreased, but the frequencies of both *P. kudriavzevii* and *C. tropicalis* increased.

The underlying risk factors in neonates with BSIs due to *P. kudriavzevii* were low birth weight (1,500 to 2,500 gm; *n* = 8), very low birth weight (1,000 to 1,500 gm; *n* = 12), extremely low birth weight (<1,000 gm; *n* = 4), preterm delivered (*n* = 20), and thrombocytopenia (*n* = 21). Age at onset of BSIs ranged from 3 to 28 days after birth. All neonates presented a nonspecific sign. All the neonates with signs and symptoms of sepsis received FLU (loading dose of 12 mg/kg of body weight, followed by 6 mg/kg of body weight) after the blood culture was collected. After the blood culture reported growth of *P. kudriavzevii*, all the neonates were treated with AMB deoxycholate for 2 weeks. Overall, follow-up data were available only for 20 of the 24 neonates: 17 were discharged with clearance of BSIs after 2 weeks of AMB-deoxycholate, and three neonates died 8–14 days after the development of BSIs.

### Genetic diversity and phylodynamic analysis of *P. kudriavzevii* strains

**Fig 1:**
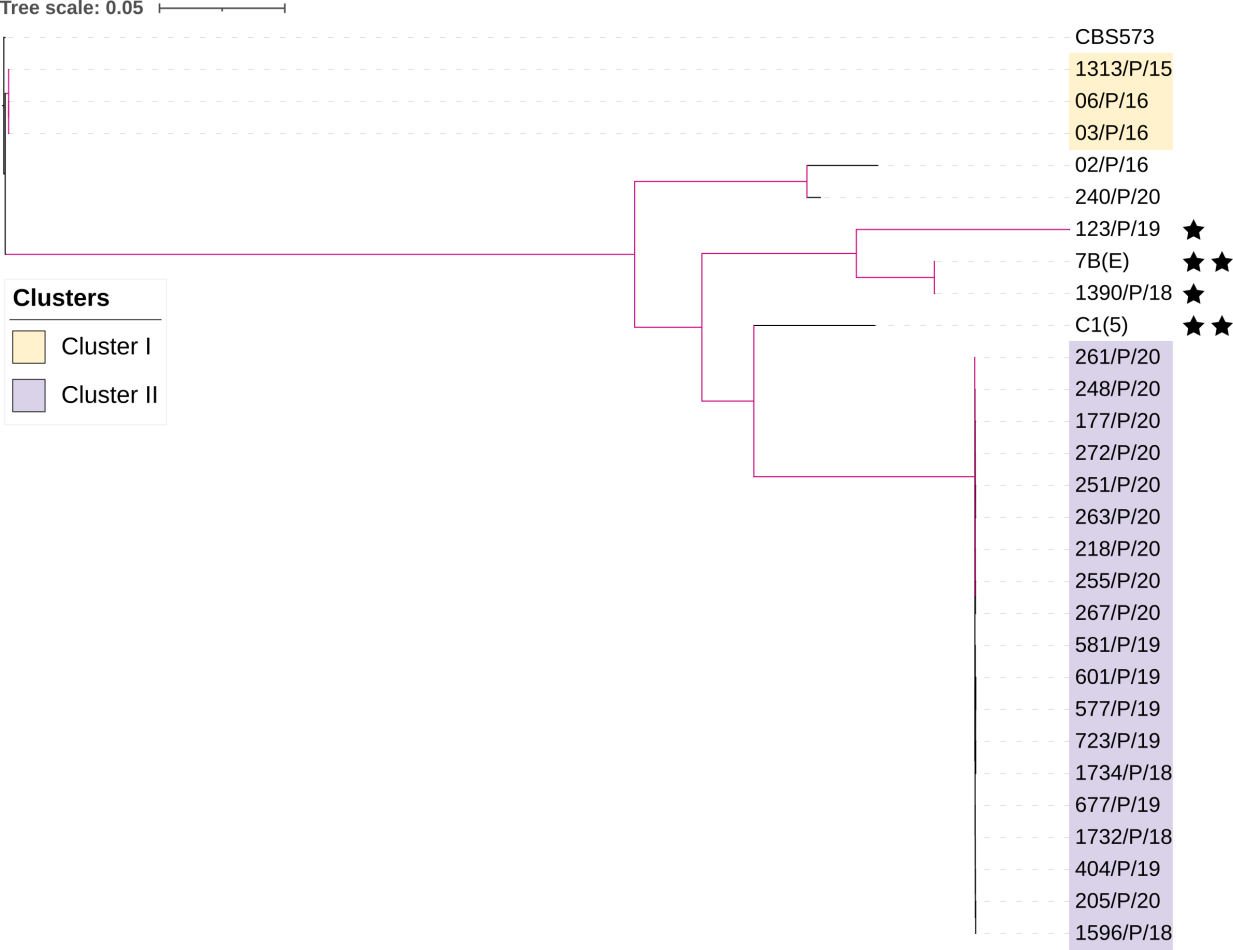
Maximum-likelihood tree depicting the relationships among 28 *P*. *kudriavzevii* strains and the reference strain CBS573. The tree was constructed using 47,982 SNP loci, with heterozygous loci represented by IUPAC degenerate nucleotide codes. The tree utilized the haplotype of the CBS573 genome at all loci as the reference. Branches with over 95% bootstrap support are highlighted in purple. ★ represents two clinical isolates, and ★★ represents two environmental isolates from other hospitals.

Single-nucleotide polymorphism (SNP) calling and phylogenetic analysis were performed for 28 strains of *P. kudriavzevii*, including 24 clinical isolates (each from a different neonate) from the same NICU, two environmental isolates from in-animate hospital environment collected previously from a different hospital, and two strains from cases of BSIs in different hospitals. These strains were compared with each other and with the reference strain CBS573 of *P. kudriavzevii*. The phylogenetic tree was constructed using 47,982 SNP sites, with heterozygous sites represented by IUPAC degenerate nucleotide codes. Pairwise SNP comparison is listed in Table S1. Overall, isolates from the NICU belonged to two major genetically distinct clusters (Cluster I; *n* = 3, and Cluster II; *n* = 19) and one small cluster of two isolates (Fig. 1). Furthermore, genomically diverse isolates, including one clinical (1390/P/18) and one environmental 7B (E) isolate each (887 SNP difference), from other hospitals was observed. Another environmental isolate C1 (5) and clinical isolate (123/P/19) from different hospitals showed divergent relationships to each other and to other strains, with 20,377 to 32,317 SNP difference with strains in clusters I and II, respectively. Of the two large clusters, Cluster I contained the reference strain, as well as three of the four NICU isolates from 2015 to 2016, whereas all NICU strains from 2018 to 2020 belong to Cluster II. Overall, strains in these two clusters were separated by 32,506 to 34,229 SNPs from each other. Within Cluster I, the three *P. kudriavzevii* isolates from 2015 to 2016 showed 48–100 SNP differences between each other. Within Cluster II, 19 *P*. *kudriavzevii* strains from 19 neonates collected during 2018–2020 formed a tight cluster. However, detailed analyses separated these 19 strains into two sub-clusters (Cluster IIa, *n* = 9; Cluster IIb, *n* = 10), with strains in these two sub-clusters differing from each other by 2,797–3,070 SNPs. Interestingly, within Sub-cluster IIa, all nine *P. kudriavzevii* isolates were from 2018 to 2019, and they differed from each other by 194–339 SNPs, whereas the 10 isolates in Cluster IIb were all *P. kudriavzevii* from 2020, and they showed 197–526 SNPs from each other. Clinical and environmental strains from other hospitals showed a large number of SNPs ranging from 20,101 to 32317 from the NICU strains.

**Fig 2:**
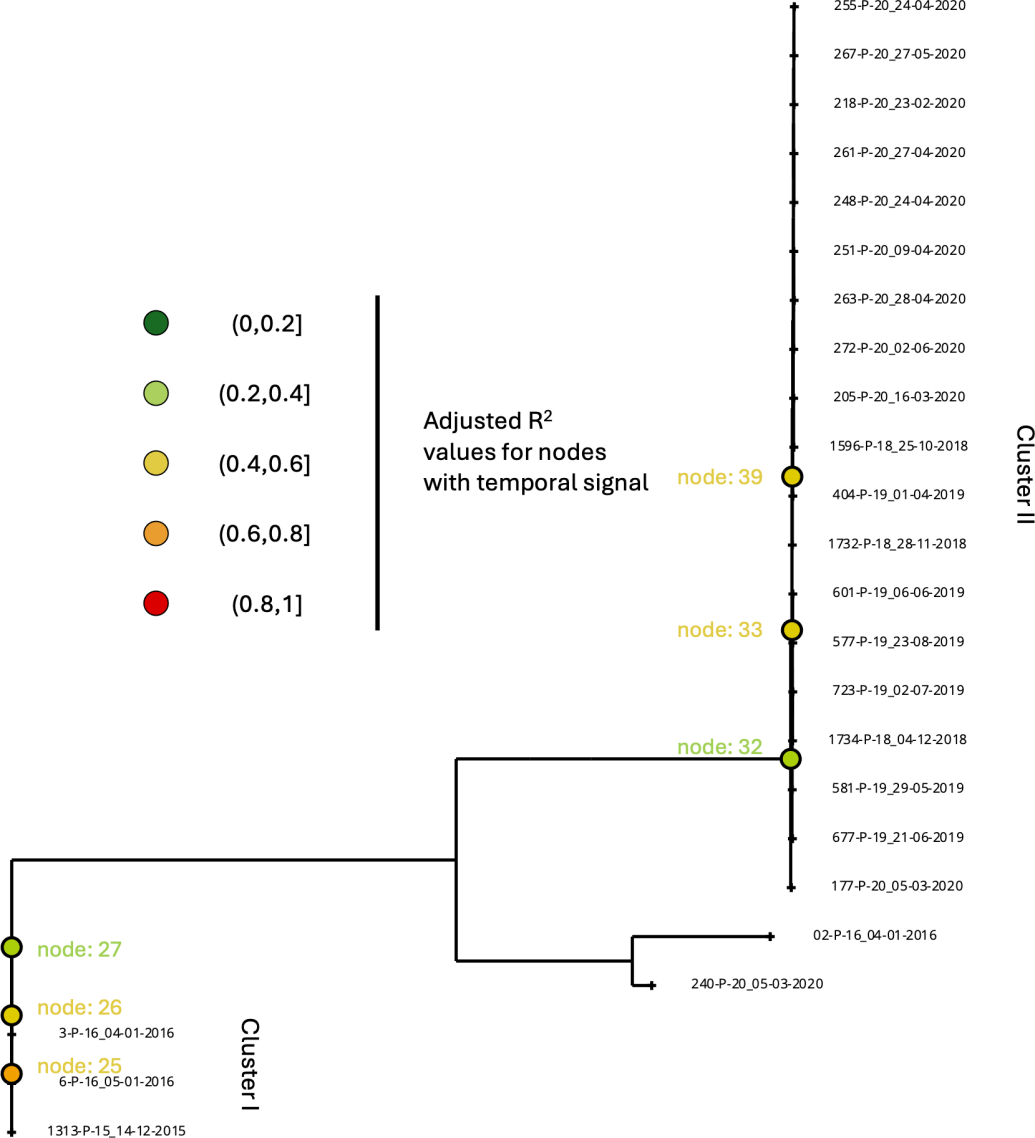
Maximum likelihood tree of 24 strains based on whole genome sequence alignment. Nodes with temporal signal are highlighted on the tree.

Furthermore, to understand the evolution of strains during the outbreak, a root-to-tip regression analysis was done by using collection dates for each clinical isolate. The root-to-tip analysis identified six ancestral nodes that exhibit a weak to moderate temporal signal, with R² ranging from 0.2 to 0.6, indicating a correlation between genetic divergence and the sampling dates (Fig. 2). These nodes belonged to two distinct clonal clusters concordant with two genetic clusters recorded in phylogenetic analysis, and nodes of each cluster were found to be ancestors of each other, suggesting a clear lineage progression overtime. Among them, node 32 was ancestral to Cluster II, and all strains within this cluster were collected from the same NICU within a 3-year period (2018–2020). The close phylogenomic relationships among strains in Cluster II are consistent with their recent shared ancestry and clonal evolution. The evolutionary rate within this cluster was estimated at 1.11 × 10^−6^ substitutions/site/year (95% confidence interval, 1.80 × 10^−7^ to 2.04 × 10^−6^). Other strains analyzed here, including the ancestor to Cluster I, showed large genetic differences among each other and most likely represented distinct ancestries predating our sampling dates that involved sexual reproduction. As a result, mutation rates estimated based on these strains would show significant upward biases. Together, the temporal and geographic clustering of these strains is consistent with the introduction and persistence of multiple distinct genotypes of *P. kudriavzevii* causing outbreaks within the same healthcare units.

#### Patterns of heterozygosity and homozygosity along chromosomes

**Fig 3:**
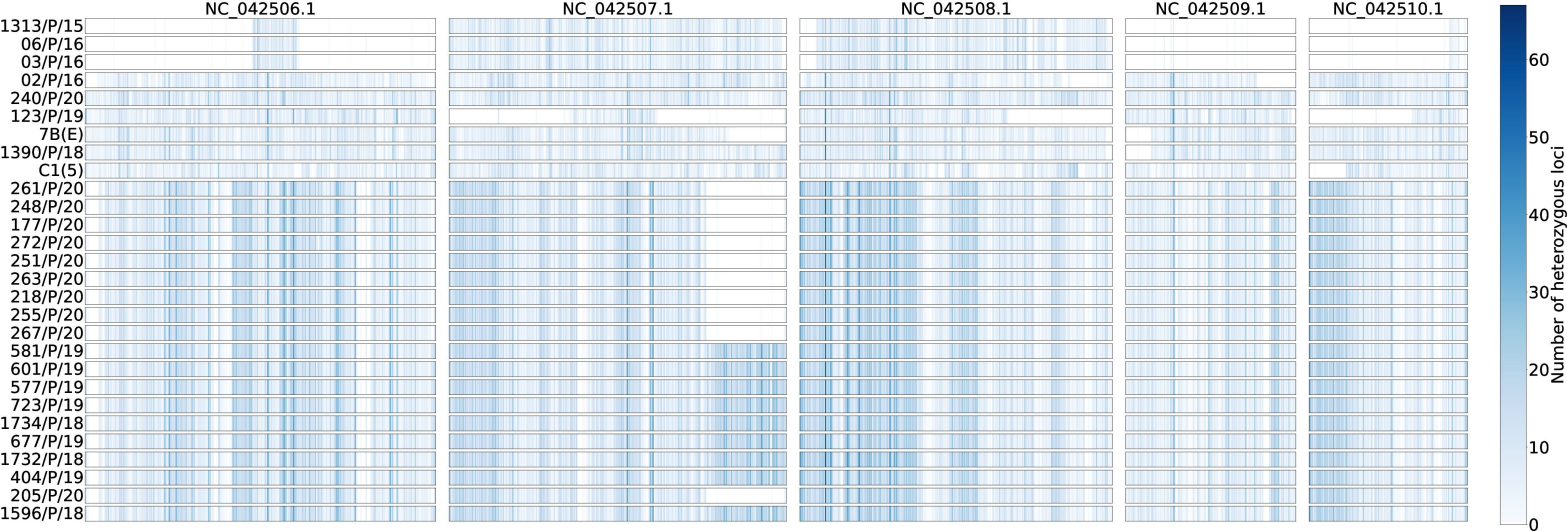
Loss of heterozygosity pattern of 28 *P*. *kudriavzevii* strains across five chromosomes. Each block illustrates the heterozygous level for a chromosome divided into non-overlapping 5,000 bp windows. The intensity of the blue color represents the number of heterozygous loci for each window ranging from 0 to 77.

**Fig 4:**
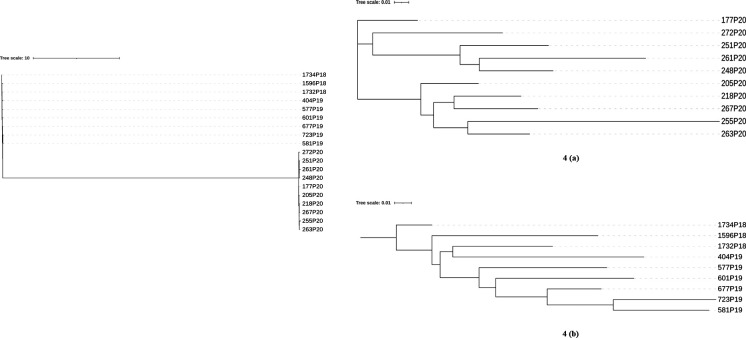
Phylogeny for *P. kudriavzevii* strains collected from 2018 to 2020 based on 1,891 LOH polymorphisms. 4(a) shows phylogenetic relationships for strains collected from 2018 and 2019 and 4(b) for strains collected from 2020 based on LOH polymorphisms. The scale bar indicates the average number of state substitutions per site.

Figure 3 shows the heterozygosity density within each strain across the five chromosomes calculated based on 5,000 bp non-overlapping windows. All strains showed a range of SNP densities across their chromosomes, from very low to very high. There are several notable observations. First, the three *P. kudriavzevii* strains from 2015 to 2016 showed very low heterozygosity on both ends of chromosome 1 (NC_042506.1) and most of chromosomes 4 (NC_042509.1) and 5 (NC_042510.1). Second, except for one region of low heterozygosity in chromosome 2 (NC_042507.1), most chromosomes in environmental strain 7B(E) showed identical heterozygosity patterns and nucleotide sequences as strain VPCI 1390/P/18 from another hospital, consistent with inter-hospital transmission. Third, there was one large region on chromosome 2 that had very low heterozygosity among the nine isolates from 2020, but this region was highly heterozygous among the 10 isolates from 2018 to 2019 (Fig. 3). This result suggests that an LOH event between 2019 and 2020 spanning this chromosomal segment was likely responsible for this observation. A phylogenetic tree based on the LOH pattern for the 2018–2020 samples is shown in Fig. 4.

#### Antifungal susceptibility data, mutations, and copy number variations in triazole resistance-related genes of outbreak strains

Overall, out of 24 NICU strains, 17% (*n* = 4) had an 8 mg/L FLU MIC value, while 83% isolates exhibited MIC > 16 mg/L (Table S2). Strains in Cluster II had a wide MIC range, from 8 to 32 mg/L. Interestingly, one isolate (VPCI 261/P/20) showed high MIC values for both FLU (16 mg/L) and ITC (2 mg/L) (Table S2). Among our 28 sequenced strains, WGS identified a total of 25 nonsynonymous mutations in 20 triazole resistance-related genes. In all outbreak strains, *ERG11* harbored a previously known missense amino acid substitution (A15V), which was observed in all tested strains, irrespective of their FLU MIC values (Table S3). Furthermore, sequencing of the *ERG11* gene in 18 strains of *P. kudriavzevii* obtained from six hospitals showed similar base substitutions, irrespective to their FLU MIC values (Table S4). Also, mutations in other 19 triazole resistance-related genes were found in both low and high FLU MIC isolates, suggesting no significant association between nucleotide substitutions in these genes and FLU susceptibility (Table S3). Notably, in seven of 19 strains in Cluster II, an increased copy number of chromosome 3 (NC_042508.1) ranging from 2.7 to 5.1 was observed. Interestingly, VPCI 261/P/20, which had the highest copy number (5.1 folds) of that of the average gene copy number, exhibited high MIC values for FLU and non-wild type for ITC (Fig. S1).

#### Fluorescence-activated cell sorting (FACS) analysis for determination of ploidy among 24 NICU strains

All tested strains of *P. kudriavzevii* had overall genome contents similar to the *Candida albicans* reference strain ATCC 90028 (Fig. S2). However, histogram of a single outbreak strain (VPCI 261/P/20) showed one additional peak to the right side of the G2 phase peak, suggesting aneuploidy in this strain.

### RNA-seq analysis of differentially expressed genes between outbreak strains belonging to different clusters

We selected one strain belonging to Cluster I (VPCI 06/P/16) and two strains from Cluster II (VPCI 218/P/20 and VPCI 261/P/20) for exploring the differentially expressed genes (DEGs) by RNA-seq analyses. These strains were selected based on their relative differences in *in vitro* susceptibility to FLU. The VPCI 06/P/16 strain from Cluster I exhibited a high FLU MIC value of 32 mg/L and a low ITC MIC value (0.125 mg/L), while the VPCI 218/P/20 from Cluster II demonstrated a relatively low FLU MIC value of 8 mg/L and a low ITC MIC value (0.5 mg/L). The multi-azole resistant strain, VPCI 261/P/20, from Cluster II showed aneuploidy, as well as high MIC values for both FLU (16 mg/L) and ITC (2 mg/L). The trimmed reads had an average mapping percentage of 95.2% with the specified reference genome of *P. kudriavzevii* using HISAT2. A total of 259 and 1607 DEGs were found in VPCI 218/P/20 and VPCI 261/P/20, respectively, when compared with Cluster I strain VPCI 06/P/16 (Fig. S3). Between the two cluster II isolates VPCI 218/P/20 and VPCI 261/P/20, 74 DEGs were identified (Table S5). The overall low number of DEGs in clusters I and II could be attributed to the limited genetic variation between the two Cluster II isolates and/or similar genetic background of *P. kudriavzevii* population analyzed in the present outbreak. Notably, the expression levels of the *ERG11* gene, the target of azole drugs in the ergosterol synthesis pathway (lanosterol 14-α-demethylase), did not show a significant difference among the three tested isolates. However, other genes involved in ergosterol biosynthesis pathway, that is, *ERG2* (*C5L36_0*A07370)*, ERG26* (*C5L36_0*A07870)*,* and *ERG25* (*C5L36_0*A11190), were significantly overexpressed in the ITC non-wild-type strain VPCI 261/P/20. Furthermore, transcription factor *UPC2* (*C5L36_0*A02350) involved in regulation of ergosterol biosynthesis was significantly upregulated in isolate VPCI 261/P/20. In contrast, no changes were observed for these genes in the VPCI 218/P/20 isolate with low FLU MIC as compared to the VPCI 06/P/16 isolate with a high FLU MIC value of 32 mg/L, as both have low ITC-MIC. The transcriptomic data suggest that alteration in *ERG2, ERG26, ERG25*, and *UPC2* in VPCI 261/P/20 might be responsible for ITC resistance in *P. kudriavzevii*. The drug efflux pumps in the ATP-binding cassette (ABC) transporter family (*ABC1* and *ABC2*) are one of the known mechanisms of azole resistance in several *Candida* spp. However, the expression of both *ABC1* and *ABC2* genes showed no difference among the three tested isolates. In the present study, we further validated the results of *ERG11*, *ABC1*, and *ABC2* in 19 blood stream isolates using real-time quantitative PCR and observed no significant overexpression of these genes in any of the high FLU MIC isolates, which further confirms the transcriptomic data (Fig. S4).

Furthermore, transcriptomic data showed that the replication factor A (*RFA1*) gene that encodes a protein involved in DNA replication and *SIR2/HST1* (C5L36_0B01610), an essential sirtuin deacetylase that regulates hyphal formation in *Candida* species, were both significantly upregulated in the VPCI 06/P/16 strain, which showed elongated filamentous cells (Fig. S5). In *C. albicans*, the depletion of members of the RFA complex like *RFA3* resulted in the formation of pseudohyphae, and deletion of *SIR2* resulted in reduced true hypha formation (18, 19). Apart from these, genes related to cell division, that is, chitin synthase III (*CHS3;* C5L36_0A03820) and cell division cycle 37 (*CDC37*; C5L36_0A01000), which is essential for cell proliferation and cell cycle progression in budding yeast, were significantly downregulated in VPCI 06/P/16. In *C. albicans*, disruption of the *CHS3* gene resulted in abnormalities in cell morphology and cell division (20). Indeed, morphological differences were observed among the tested *P. kudriavzevii* strains by scanning electron microscopy, where cells of strain VPCI 06/P/16 showed more abundant mycelial filaments than cells of VPCI 261/P/20 (Fig. S5).

## DISCUSSION

This study analyzed the genomic epidemiology of BSIs due to *P. kudriavzevii* in a single NICU. Over a period of 5 years, *P. kudriavzevii* strains in this NICU showed marked genomic diversity. In fact, genomic analysis suggested three distinct outbreaks of BSIs, including cases ranging from three to 10 neonates during the 5-year span. Interestingly, the first episode involved three neonates spanning a period of 1 month (December 2015 to January 2016) who were infected with clonal strains of *P. kudriavzevii* showing marked LOH at chromosomes 1, 4, and 5. Intriguingly, the second outbreak occurred in 2018–2019 involving nine cases. All nine neonates were infected by genetically very similar strains of *P. kudriavzevii*. The third outbreak involving 10 neonates occurred in a period of 5 months in 2020 and caused by strains very similar to those causing the second outbreak but with a large LOH at chromosome 2. Furthermore, the root-to-tip regression analysis of NICU isolates suggested that the strains from 2018 to 2020 emerged from strains belonging to 2015–2016. The circulation of two separate strain clusters of *P. kudriavzevii* in 2015–2016 and 2018–2020 suggests nosocomial transmission in the NICU in different time periods. Suboptimal infection control practices may have contributed to the transmission of strains among neonates. Outbreaks of healthcare-associated *P. kudriavzevii* BSIs have been described in recent years (6, 13, 14, 16, 21). In these outbreak investigations, conventional molecular techniques, such as random amplified polymorphic DNA analysis, restriction endonuclease analysis of genomic DNA, and fluorescent amplified fragment length polymorphism, were used to infer the relatedness of strains (6, 13, 14, 16, 21). However, the techniques often have issues with limited reproducibility and/or low discrimination power. Other molecular markers, such as microsatellite analysis, multi-locus sequence typing (MLST), and WGS, have become increasingly used for genotyping human fungal pathogens and for investigating outbreaks. Recently, a strain typing method based on a set of short tandem repeat sequences was established for *P. kudriavzevii* and found to have comparable discrimination power among strains as genome-wide SNPs (22). In the present study, genomic surveillance detected clustering of cases retrospectively; thus, the environmental sampling to ascertain the source was not undertaken. Nevertheless, the emphasis of routine surveillance to detect concerning nosocomial transmission is warranted for preventing or containment of the outbreaks.

WGS-based investigations have been carried out on model lab strains of *P. kudriavzevii* (81-B-5, CBS573T, 129, CBS5147T) and 30 clinical and environmental isolates (23–25). Those clinical strains were collected from hospital laboratories of China, Europe, Sri Lanka, Argentina, and Brazil and included mainly sputum, urine, vaginal, fecal, and blood samples. Phylogenetic analysis of SNPs showed no clear separation between clinical and environmental isolates, suggesting that *P. kudriavzevii* infections were likely independently acquired from various environments (25). Furthermore, genomic analysis of clinical and environmental strains showed that loss of heterozygosity is common in *P. kudriavzevii* occurring in 30 of 32 strains examined (25). Importantly, in the present study, all strains showed heterogenous distributions of heterozygous SNPs among their chromosomes. This pattern could have been derived from either sexual reproduction or asexual reproduction through mitotic recombination and/or gene conversion. Indeed, *P. kudriavzevii* is capable of sexual reproduction in the lab. However, the overall similar heterozygosity pattern among strains, especially those 19 strains from the NICU, suggested that the LOH events most likely occurred through mitotic recombination and gene conversion during asexual reproduction. Specifically, meiosis and sexual mating would generate a diversity of recombinants each with its unique regions of heterozygosity and homozygosity throughout each chromosome and the entire genome within each recombinant. In contrast, each mitotic recombination/gene conversion typically impacts a unique portion of the genome while maintaining the heterozygosity patterns for the remaining parts of the genome.

Aside from contributing to rapid genetic change among strains within a clonal cluster, LOH can also influence the temporal pattern of relationships among the strains, as reflected by the heterogenous adjusted R^2^ values across our samples. For example, a single LOH of a large chromosomal segment (or a whole chromosome) can cause a substantial genetic difference between strains, resulting in a significant deviation of temporal relationships among strains based on a steady accumulation of mutation model. Most evolutionary dating programs assume a consistent mutation rate (molecular clock) and no meiotic or mitotic recombination. *P. kudriavzevii* is capable of both meiotic recombination and mitotic recombination, such as LOH. Indeed, we inferred different numbers of LOH events involving different chromosomal segments for both clusters I and II (Fig. 4), and their R^2^ values varied among groups of strains. Other possibilities causing inconsistent temporal relationships among strains include introduction of new strains and thus new genetic variations to the analyzed population and incomplete sampling. Specifically, some of the strains we sampled and analyzed may not represent the true spatial and temporal populations of *P. kudriavzevii* in the NICU very well.

Interestingly, the isolate with high MIC values for both FLU and ITC was aneuploid and had large LOH at chromosome 2, as well as increased copy numbers of chromosome 3. In *C. albicans*, both losses of heterozygosity and aneuploidy were associated with triazole resistance. Similar mechanisms might also be operating in *P. kudriavzevii*. Loss of heterozygosity is common in other diploid yeasts, such as *S. cerevisiae*, *C. albicans*, and *C. tropicalis*, to restore DNA damage or diploidy during reproduction (26, 27). Loss of heterozygosity events can be induced by stress, notably several stressors, including exposure to heat, drugs, an oxidizing agent, such as hydrogen peroxide, and FLU in *C. albicans* and *Cryptococcus neoformans* (28, 29). A recent study showed the rapid *in vitro* development of resistance in *P. kudriavzevii* strains exposed at low concentrations of FLU (4 mg/mL) and VRC (0.06 mg/mL) for 30 days, attributing to the intrinsic resistance of *P. kudriavzevii* to FLU (30). Indeed, long-term usage of FLU in our clinical settings may have selected LOH events and adaptation in *P. kudriavzevii* population in the NICU.

In this study, 87.3% (*n* = 144) of isolates had high MICs for FLU (MIC range of 16–32 mg/L), and no resistance to VRC and echinocandins were observed. Previously, the global SENTRY Antimicrobial Surveillance Program reported 95% *P. kudriavzevii* isolates to be VRC-susceptible and a very low echinocandin resistance (0.0–1.7%) among 421 isolates during 2006–2016 (3). Low affinity of *ERG11* and overexpression of *ERG11, ABC1*, and *ABC2* gene are a well-known mechanism in *P. kudriavzevii* that has been associated with acquired azole resistance (30–33). Nonetheless, the mechanisms responsible for innate FLU-R in *P. kudriavzevii* are believed to be the low affinity of Erg11, high-level expression of the multidrug efflux pump Abc1p, and presence of tandem ABC11–ABC1 gene pair at the ABC11–ABC1 locus (25, 34, 35). In *P. kudriavzevii*, the ABC11–ABC1 locus at chromosome 4 generally contains tandem *ABC11* and *ABC1* gene pair, but due to ectopic recombination between *ABC1* and *ABC11* genes, either it forms one gene (an *ABC11-1* chimera) or is expanded to three genes (36). Recently, Douglass et al. reported that eight of 34 strains were heterozygous at the ABC11–ABC1 locus, containing a single fused gene (*ABC11-1* chimera) at one chromosome. Interestingly, four of five FLU susceptible strains were heterozygous and showed the presence of single gene at the ABC11–ABC1 locus (25). Here in our study, we did not find a significant role of *ERG11* and *ABC* transporter genes in reduced FLU susceptibility. Instead, the transcriptomic data suggest that FLU resistance in *P. kudriavzevii* might not be associated with altered expression of ergosterol biosynthesis pathway genes and transcription factor *UPC2*. In addition, several genes involved in cell division and filamentation in yeast (i.e., *CHS3*, *RFA1*, *CDC37*, and *SIR2)* were dysregulated in the high FLU MIC Cluster I isolate VPCI 06/P/16, indicating that these altered morphological features are likely associated with drug resistance and adaptation to environmental stresses in *P. kudriavzevii*. Together, our analyses indicate that *P. kudriavzevii* is an important nosocomial pathogen capable of rapid evolution and persistence in hospital environments and causing blood stream infections in hospitalized neonates with an immature immune system.

## MATERIALS AND METHODS

### Isolate identification and antifungal susceptibility testing

Over the course of 8 years from 2015 to 2023, 165 clinical isolates of *P. kudriavzevii* were collected for an ongoing antifungal resistance surveillance from 16 tertiary care hospitals in Delhi and the adjoining National Capital Region. The isolates were mainly from blood (*n* = 132), followed by urine (*n* = 15), upper respiratory tract (*n* = 14), and vagina (*n* = 4). All 165 isolates were stored at −70°C and screened on CHROMagar *Candida* medium (Becton Dickinson, Baltimore, MD, USA), followed by sub-culture on Sabouraud’s dextrose agar (SDA) at 37°C for 24 h. The species identification was done by matrix-assisted laser desorption ionization–time of flight mass spectrometry (Bruker Biotyper OC version 3.1; Daltonics, Bremen, Germany) with a score value of >2 using formic acid extraction method as per instruction of the manufacturer (37).

#### *In- vitro* antifungal susceptibility testing

Antifungal susceptibility of all isolates was tested against nine antifungal drugs using the broth micro dilution method, as recommended by CLSI M27-A3 (38). The tested drugs include five azoles, namely, FLU (Sigma, St. Louis, MO, USA), ITC (Lee Pharma, Hyderabad, India), posaconazole (POS, Merck, Whitehouse Station, NJ, USA), VRC (Pfizer, Groton, CT, USA), and ISA (Basilea Pharmaceutical, Basel, Switzerland), two echinocandins, namely, micafungin (MFG, Astellas, Toyama, Japan), and AFG (Pfizer), AMB (Sigma) and 5-FC (Sigma). The dilution ranges for nine antifungal drugs were as follows: 0.03–16 mg/L for ITC, VRC, 5-FC, and AMB; 0.015–8 mg/L for ISA, POS, and two echinocandins; and 0.25–128 mg/L for FLU. *Candida krusei* ATCC 6258 and *Candida parapsilosis* ATCC 22019 were used as reference strains. The MIC of all isolates with low FLU MIC values and outbreak strains were repeatedly tested 10 times, and the median MIC values were calculated. Statistical parameters, including GM, median MIC, MIC range, MIC_50_, and MIC_90_, were calculated by using Prism version 6.00 (GraphPad software). In accordance with the CLSI M27M44S 3rd edition, no breakpoints for FLU for *P. kudriavzevii* have been assigned. We categorized the isolates in two sets based on FLU MIC values (i.e., isolates with MIC range 16–32 mg/L were placed in the high-FLU MIC group, and isolates with MIC value ≤ 8 mg/L were categorized in low FLU MIC group. For ITC, the epidemiological cut-off value of ≥1 mg/L was defined as non-wild type or resistant (39).

### Whole-genome sequencing and phylogenetic analysis of *P. kudriavzevii* strains from NICU

We sequenced the genomes of 28 isolates, including 24 bloodstream isolates obtained from neonates and two environmental isolates from the floor of a different hospital using the NOVASEQ 6000 Sequencer (San Diego, USA). Additionally, two bloodstream isolates that differed significantly in their FLU MICs from another hospital (isolates VPCI 123/P/19 and VPCI 1390/P/18 with 4 and 32 mg/L MIC values, respectively) were selected for WGS.

For each isolate, DNA extraction was carried out using the DNeasy Ultraclean Microbial Kit (Thermo Fisher, MA, USA). The extracted DNA was quantified using NanoDrop 1000. Whole-genome sequence libraries were prepared using the NEBNext Ultra II DNA FS Kit (New England Biolabs, Ipswich, MA, USA). The reference strain CBS573 genome was included for SNP calling and phylogenetic analysis. First, reads of each strain were mapped to the *P. kudriavzevii* reference genome CBS573 (ASM305444v1) using BWA v 0.7.17 (40). Alignments were then converted to BAM format and sorted by chromosomes using SAMtools v 1.13 (41). Duplicated reads were labeled using Picard v 2.26.3 (42). Genomic variants for individual strains were identified using GATK v 4.2.5 HaplotypeCaller in GVCF mode. GVCF files were merged and genotyped using CombineGVCFs and GenotypeGVCFs, respectively. Afterwards, SNPs were extracted from the merged GVCF file using Select Variants and filtered using Variant Filtration with the following hard filters, QD < 2.0, FS > 60.0, SOR > 3.0, MQ < 40.0, MQRankSum < −12.5, and ReadPosRankSum < −8.0. Annotation was conducted using SnpEff v5.0 (43). We deposited FASTQ files for all isolates at GenBank under BioProject no. PRJNA108400.

#### Phylogenetic analysis

To construct the phylogenetic tree, SNP loci with missing genotype calling in over 50% of the samples were removed using VCFtools v 0.1.16 (44), and the filtered loci were concatenated into SNP sequences using vcf2phylip v2.0 (45), with CBS573 as the reference. A maximum-likelihood tree was constructed using IQ-tree v2.0.7 (46) with 500 bootstrap replicates.

For the phylodynamic analysis, a root-to-tip regression analysis was performed using the program, Phylostems v0.1.1 (47). The input file is a maximum phylogenetic tree generated based on whole genome and the sampling dates of the 28 isolates. For the tree construction, whole genome sequence alignments were constructed based on the SNP information and reference genome. Heterozygous loci were represented using degenerate nucleotide codes. Then, a maximum phylogenetic tree was generated using IQtree v2.0.7 (46) with 1,000 ultrafast bootstrap replicates. The best-fit substitution model was determined using ModelFinder. Tree was rooted using iTOL v5 (48).

#### Loss of heterozygous analysis

*P. kudriavzevii* is a diploid yeast capable of sexual reproduction. Within each strain at each locus/nucleotide site, there are two alleles/nucleotides. A strain with the same allele/nucleotide at the same site is called a homozygote at the locus/site, while that with different alleles/nucleotides is called a heterozygote. During both sexual and asexual reproduction, heterozygosity could be lost due to meiotic and mitotic recombination. Heterozygosity could also be gained from sexual mating and the emergence of new mutation during reproduction. In this study, we analyzed the heterozygosity pattern across the genomes for all sequenced strains. Each chromosome was divided into 5,000 bp non-overlapping windows, and the number of heterozygous loci/nucleotide sites was counted for each window. The heterozygosity pattern was plotted for individual strains across the five reference chromosomes using Python v3.11.4 matplotlib v3.7.1 (49).

#### Antifungal related genes and mutations

We screened mutations in genes, which have been reported to be associated with antifungal resistance, including *ERG* family genes, *FUR1*, *MDR1*, *MSH2*, *CDR1*, *PMS1*, *PDR1*, *HOG1*, *YMC1*, and *MLH1,* following protocols described previously (37).

#### Copy number variation

In addition, the copy numbers of these genes within individual strains were assessed based on read depth. Copy number variation (CNV) analysis was conducted using CNVpytor v1.3.1 (50), with bin size set as 1,000 bp. CNV results were merged over the 28 samples. The following parameters were set to filter low-quality calls: Q0-range: [−1.0, 0.5], p-range: [0.0, 0.0001], size_range: [5000, inf], and dG_range: [10000, inf]. CNV calls were merged for 28 samples.

#### Ploidy analysis of *P. kudriavzevii* strains

All 24 *P*. *kudriavzevii* bloodstream isolates obtained from the NICU were subjected to FACS (FACSAria III; BD Biosciences, USA) to assess their ploidy. *Candida glabrata* ATCC 15545 (haploid control) and diploid *C albicans* ATCC 90028 were used as a reference strains. Sample preparation and analysis were done, as described previously (37). FlowJo_v10.10.0 software was used to generate the FACS layout diagram.

### RNA-seq analysis

A total of three outbreak strains, one belonging to Cluster I with high FLU MIC value (VPCI 06/P/16; 32 mg/L; ITC MIC value 0.125 mg/L) and two strains from Cluster II, that is, one with low FLU MIC value (VPCI 218/P/20; 8 mg/L; ITC MIC value 0.5 mg/L) and another aneuploidy strain (VPCI 261/P/20) with 16 and 2 mg/L MIC values for FLU and ITC, respectively, were analyzed in triplicate.

Briefly, fresh (after 17 h of incubation) *P. kudriavzevii* cultures were inoculated into yeast peptone dextrose (YPD, initial OD_600_ of 0.1) broth and grown at 37°C for 4 h. The total RNA was extracted and purified using the Genejet RNA Purification Kit (Thermo Fisher Scientific, USA). The RNA samples were quantified using a Qubit 2.0 fluorometer (ThermoFisher Scientific, USA), and RNA integrity was checked using TapeStation (Agilent Technologies, Palo Alto, CA, USA). The RNA sequencing libraries were prepared using the NEBNext Ultra II RNA Library Preparation Kit following the manufacturer’s instructions (New England Biolabs, Ipswich, MA, USA). Briefly, mRNAs were initially enriched with Oligo-d(T) beads. Enriched mRNAs were fragmented for 15 min at 94°C. First- and second-strand cDNAs were subsequently synthesized. cDNA fragments were end-repaired and adenylated at 3′ends, and universal adapters were ligated to cDNA fragments, followed by index addition and library enrichment by PCR with limited cycles. The sequencing libraries were validated on the Agilent TapeStation (Agilent Technologies, Santa Clara, California, USA) and quantified by using a Qubit 2.0 fluorometer (Thermo Fisher Scientific, USA), as well as by quantitative PCR (KAPA Biosystems, Wilmington, MA, USA). The sequencing libraries were multiplexed and clustered onto a flow cell. After clustering, the flow cell was loaded onto the Illumina HiSeq instrument according to the manufacturer’s instructions. The samples were sequenced using a 2 × 150 bp paired end configuration. Three biological replicates for each strain were sequenced.

After investigating the quality of the raw data, sequence reads were trimmed to remove the adapter sequences and nucleotides with poor quality using Trim Galore v0.4.5 (https://github.com/FelixKrueger/TrimGalore). The trimmed reads were mapped to the reference genome of the CBS573 strain using the HISAT2 with default settings (51). BAM files were generated as a result of this step. Unique gene hit counts were calculated by using FeatureCounts (52). Only unique reads that fell within exon regions were counted. The read counts were normalized, and EdgeR differential gene expression analysis was done using DEBrowser V1.30.2 (https://debrowser.umassmed.edu/) (53). The Exact test was used to generate *P* values for differential abundance for each gene between two samples and log2 fold changes. Genes with adjusted *P* values < 0.05 and absolute log2 changes > 0.585 (1.5-fold change) were called as differentially expressed genes for each comparison. Normalized read counts were used for PCA in DEBrowser (53). Heat map of hierarchical clustered and differentially expressed genes was generated using data mining tool, Orange3.37.0 (54). Hierarchical clustering used the Euclidean distance and average cluster linking. The resulting dendrograms show the degree of similarity in gene expression. Venn diagrams were generated using Venny 2.1 (http://bioinfogp.cnb.csic.es/tools/venny) (55). For further downstream analysis, *Saccharomyces cerevisiae* orthologs were retrieved from the *P. kudriavzevii* annotation file and depicted the interested genes in volcano plots.

### Real-time quantitative PCR to confirm gene expression

To validate transcriptomic results and investigate the role of *ERG11* and transporter genes (*ABC1* and *ABC2*) in *P. kudriavzevii*, transcript levels of a set of 19 isolates exhibiting 4- to 8-fold differences in the MICs of FLU were analyzed by RT-qPCR using the ABI Prism 7500 Fast System (Applied Biosystems, CA, USA). Twelve of the 19 strains had relatively low MIC values between 4 and 8 mg/L, and the remaining seven had relatively high MIC values of 16–32 mg/L. For this analysis, all isolates were grown in YPD broth at 37°C for 16 h at 200 rpm. The secondary culture was prepared by inoculating 1 × 10^8^ cells (OD_600_ 0.3) from 16 h culture and further incubated at 37°C at 200 rpm for 3 h (mid log phase). After 3 h, total RNA was extracted by using TRIzol reagent (Sigma) and stored at −20°C. Purity of extracted RNA was checked to ensure a OD_260_/OD_280_ absorption ratio of >1.95. Subsequently, RNA was treated with RNase-free DNase I (NEB, MA, USA), and the purified RNA was used for the synthesis of cDNA using a High-capacity cDNA Reverse Transcription Kit (Applied Biosystems) with random primers. Real-time PCR was performed for the amplification of endogenous reference gene *ACT1* and target genes *ERG11*, *ABC1*, and *ABC2*, as described previously (31, 56).

The reaction mixture contained 1× Power SYBER Green PCR Master Mix (Thermo Fisher Scientific, MA, USA), sense and antisense primers, 2 µL cDNA, and RNase-free water at a final volume of 25 µL. Samples were subjected to initial holding stage at 50°C for the 20 s and 95°C for 10 min, followed by 35 cycles of 15 s at 95°C and 1 min at 60°C. Annealing and extension steps were undertaken at 60°C, and fluorescence data were analyzed with the 7500 software v2.0.6 (Applied Biosystems). The expression of target genes (i.e., *ERG11*, *ABC1*, and *ABC2*) in strains with MIC ≥ 16 mg/L was evaluated relative to that in the low MIC strain (VPCI 1619/*P*/16, 4 mg/L). The expression level was calculated by using the 2^−ΔΔCT^ analysis method. The samples were processed in triplicates, and a ≥2-fold change in expression was considered a significant change. The two-tailed Student’s *t*-test was used to compare categorical variables. *P*  <  0.05 was considered statistically significant.

### PCR amplification and sequencing of the *ERG11* gene

To confirm mutations obtained through WGS at the *ERG11* gene, 18 *P*. *kudriavzevii* strains representing a range of FLU MICs (4–32 mg/L) were sequenced at the *ERG11* loci using the Sanger sequencing method. Briefly, genomic DNA extraction was performed by using the chloroform–isoamyl alcohol method, as described by (57). The *ERG11* gene sequence of *Pichia kudriavzevii* strain 72PhC (GenBank accession no. KY967263.1) was downloaded from the National Center for Biotechnology Information and used for primer designing. Primers targeting *ERG11* (Ckr*ERG11*_F, 5′-GCCGATAATACTCGTTGCGA-3′, Ckr*ERG11*_R 5′-GGAAAGAAAGGGAAAACACTGT-3′, Ckr*ERG11*_1F, 5′-GTGTTTTCCTTTCTCTTGTTGG-3′, Ckr*ERG11*_1R, 5′-ACATAATGGCCCTTTGGAAC-3′) were designed using the Primer 3 program. PCR amplification of the *ERG11* sequence was carried out in 50 µL final reaction volume using a Taq Polymerase Kit. Thermocycler (Thermo Fisher Scientific, USA) conditions were as follows: denaturation for 3  min at 95°C, followed by 35 cycles of 30 s at 95°C for denaturation, 30 s at 54°C for annealing, 180 s at 72°C for elongation, and a final incubation for 10 min at 72°C. High-prep DTR Purification Kit (Roche Diagnostics, Madrid, Spain) was used for the purification of PCR products. The purified product was examined using 1% agarose gel electrophoresis, followed by staining with ethidium bromide and visualized using Molecular Imager Gel Doc (Bio-Rad). Sequencing PCR was performed by using Big Dye Terminator Kit v3.1 (Applied Biosystems CA, USA) in a 10 µL reaction volume having a PCR primer at 2.5 mM concentration. Sequenced PCR products were purified with the help of the High-prep DTR Purification Kit as per manufacturer’s instructions and finally analyzed on an ABI3130Xl Genetic Analyzer (Applied Biosystems). BioEdit software (version 7.0.5.3) was used to get consensus sequences. *ERG11* gene sequences of all isolates were aligned against the *ERG11* sequence of *P. kudriavzevii* strain CBS573 (GenBank accession number: NC_042509.1), as described previously (25).

### Scanning electron microscopy

To evaluate potential micro-morphological variation between the two isolates, including one strain belonging to Cluster I (VPCI 06/P/16) and another strain from Cluster II showing evidence of aneuploidy (VPCI 261/P/20), scanning electron microscopy was done following a protocol described previously (37). Briefly, a single colony of each strain was subcultured on SDA, followed by inoculation into the YPD broth incubated at 37°C for 17 h in a shaking incubator at 200 rpm. An inoculum with 1 × 10^8^ cells (OD600 = 0.1) from overnight culture was transferred in YPD broth, and cells were fixed with 2.5% glutaraldehyde (Sigma-Aldrich) in phosphate-buffered saline (PBS; pH 6; Sigma-Aldrich) for 16 h at 4°C. After primary fixation, cells were washed twice with PBS, and then fixed with 1% osmium tetroxide for 3 h at 4°C. The fixed cells were dehydrated by using increasing concentrations of ethanol from 30 to 100%, followed by drying. The samples were further sputter coated with gold (JEC 300 sputter coater) and analyzed under a scanning electron microscope (JEOL JSM 6610LV; Japan) with a ×4,000 magnification.

## Data Availability

The raw RNA-seq data have been deposited to the Gene Expression Omnibus (GEO) under the accession number GSE282019.

## SUPPLEMENTAL MATERIAL

Fig. S1 aac.01709-24-s0001.pdf Read depth plot for 28 *P. kudriavzevii* strains across region NC_042509.1:613002-642999.

Fig. S2 aac.01709-24-s0002.docx Histograms representing DNA content or cell cycle profile obtained by fluorescence-activated cell sorting.

Fig. S3 aac.01709-24-s0003.pdf Heat map, Venn diagram, and volcano plots.

Fig. S4 aac.01709-24-s0004.pdf Relative gene expression of ERG11, ABC1, and ABC2 in seven isolates with high fluconazole MIC value of 16-32 mg/L and 12 isolates with low FLU MIC 4–8 mg/L.

Fig. S5 aac.01709-24-s0005.pdf Scanning electron micrograph of *P. kudriavzevii*.

Table S1 aac.01709-24-s0006.xlsx Pairwise SNP comparison among 28 strains of *P. kudriavzevii* and one reference strain.

Table S2 aac.01709-24-s0007.docx Antifungal susceptibility profiles of 24 *P. kudriavzevii* strains.

Table S3 aac.01709-24-s0008.xlsx Nonsynonymous mutations in 20 triazole resistance-related genes in *P. kudriavzevii* strains by WGS.

Table S4 aac.01709-24-s0009.docx *In vitro* susceptibility profile and point mutations in ERG11 gene of *P. kudriavzevii* strains obtained from patients admitted in the intensive care units of six hospitals.

Table S5 aac.01709-24-s0010.xlsx Differentially expressed genes in two isolates belonging to cluster II, i.e., VPCI 218/P/20 and VPCI 261/P/20, exhibiting FLU-MIC 8 mg/L and 16 mg/L, respectively, with respect to isolate from cluster II (VPCI 06/P/16) with high FLU-MIC value of 32 mg/L.

ASM does not own the copyrights to Supplemental Material that may be linked to, or accessed through, an article. The authors have granted ASM a non-exclusive, world-wide license to publish the Supplemental Material files. Please contact the corresponding author directly for reuse.
