# Supplementary material for: Genomic analyses reveal high diversity and rapid evolution of Pichia kudriavzevii within a neonatal intensive care unit in Delhi, India
Source: Antimicrob Agents Chemother. 2025 Jan 24;69(3):e01709-24. doi: 10.1128/aac.01709-24 (PMC11881565; doi:10.1128/aac.01709-24)
Supplement: Fig. S2 — Histograms representing DNA content or cell cycle profile obtained by fluorescence-activated cell sorting. [file aac.01709-24-s0002.docx]

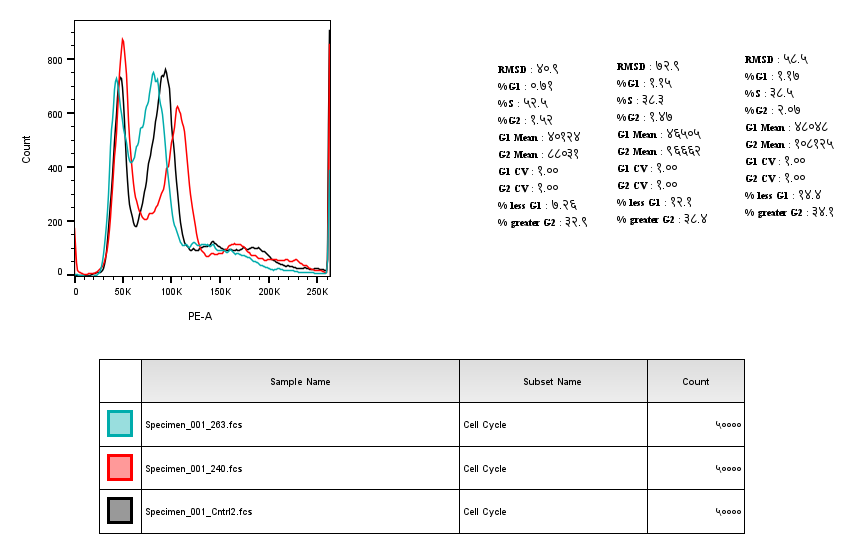


**Figure S2:** Histograms representing DNA content or cell cycle profile obtained by fluorescence-activated cell sorting. For comparison, a diploid *C. albicans* ATCC90038 indicated by black color was used as reference strains. Strain VPCI 06/P/16 from cluster I had diploid genome content highlighted by blue color. Strain from cluster II VPCI 261/P/20 represented by red color showed a shift of the G2 peak to the right, consistent with aneuploidy. The x-axis represents nuclear fluorescence, and y-axis represents cell number.
