# Supplementary material for: Genomic analyses reveal high diversity and rapid evolution of Pichia kudriavzevii within a neonatal intensive care unit in Delhi, India
Source: Antimicrob Agents Chemother. 2025 Jan 24;69(3):e01709-24. doi: 10.1128/aac.01709-24 (PMC11881565; doi:10.1128/aac.01709-24)
Supplement: Fig. S3 — Heat map, Venn diagram, and volcano plots. [file aac.01709-24-s0003.pdf]

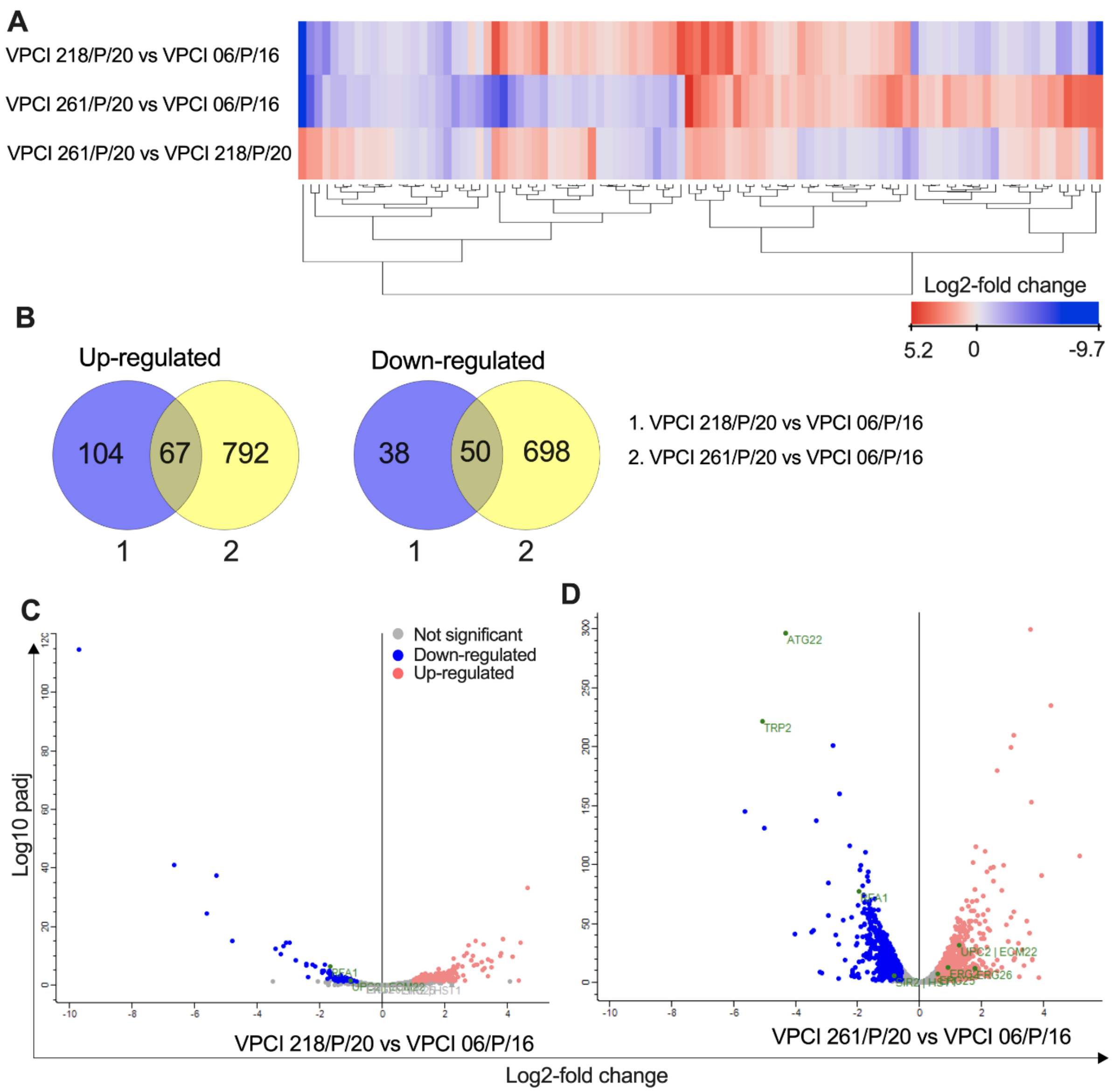

Figure S3: A. Heat map of hierarchical clustering and differentially expressed genes in three *P. kudriavzevii* outbreak isolates VPCI 06/P/16 (cluster I; FLU-MIC 32 mg/L), VPCI 218/P/20 (cluster II, FLU MIC 8 mg/L) and an aneuploid strain VPCI 261/P/20 (cluster II; FLU-MIC 16 mg/L, ITC-MIC 2 mg/L). Log2-fold change (FC) expression values are color-coded according to the legend on the bottom. B. Venn diagram showing the number of differentially expressed genes based on cut off value (≥1.5-fold change) (padj ≤ 0.05). C and D. Volcano plot demonstrate the number of significantly DEGs. Every dot represents a differentially expressed gene. Gray dots were non-significantly different genes. The red colored dots represent significantly upregulated genes and blue represent significantly downregulated genes.
