# Supplementary material for: Genomic analyses reveal high diversity and rapid evolution of Pichia kudriavzevii within a neonatal intensive care unit in Delhi, India
Source: Antimicrob Agents Chemother. 2025 Jan 24;69(3):e01709-24. doi: 10.1128/aac.01709-24 (PMC11881565; doi:10.1128/aac.01709-24)
Supplement: Fig. S4 — Relative gene expression of ERG11, ABC1, and ABC2 in seven isolates with high fluconazole MIC value of 16-32 mg/L and 12 isolates with low FLU MIC 4–8 mg/L. [file aac.01709-24-s0004.pdf]

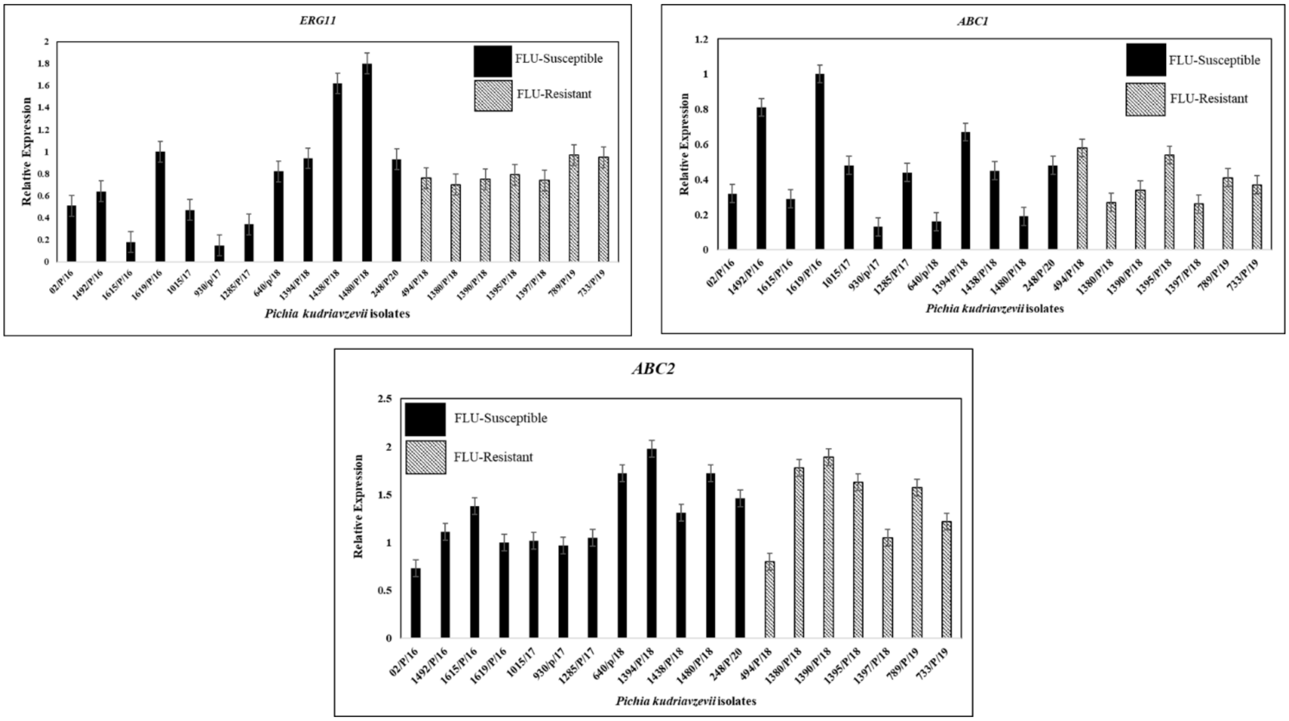

Figure S4: Relative gene expression of *ERG11*, *ABC1*, and *ABC2* in seven isolates with high fluconazole MIC value of 16-32 mg/L and twelve isolates with low FLU-MIC 4-8 mg/L. Relative expression of each gene in *P. kudriavzevii* were quantified and normalized relative to housekeeping gene *ACT1*.
