# Supplementary material for: Genomic analyses reveal high diversity and rapid evolution of Pichia kudriavzevii within a neonatal intensive care unit in Delhi, India
Source: Antimicrob Agents Chemother. 2025 Jan 24;69(3):e01709-24. doi: 10.1128/aac.01709-24 (PMC11881565; doi:10.1128/aac.01709-24)
Supplement: Fig. S5 — Scanning electron micrograph of P. kudriavzevii. [file aac.01709-24-s0005.pdf]

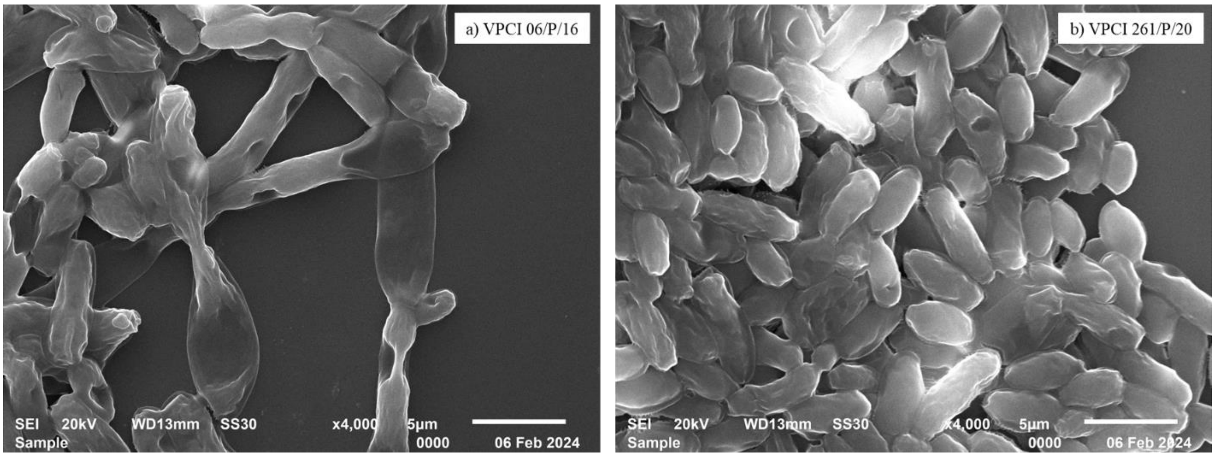


**Figure S5:** Scanning electron micrograph of *P. kudriavzevii*. a) VPCI 06/P/16 and b) VPCI 261/P/20 strains belongs to cluster I and II respectively from the NICU.
