# Supplementary material for: Genomic analyses reveal high diversity and rapid evolution of Pichia kudriavzevii within a neonatal intensive care unit in Delhi, India
Source: Antimicrob Agents Chemother. 2025 Jan 24;69(3):e01709-24. doi: 10.1128/aac.01709-24 (PMC11881565; doi:10.1128/aac.01709-24)
Supplement: Table S2 — Antifungal susceptibility profiles of 24 P. kudriavzevii strains. [file aac.01709-24-s0007.docx]

**Table S2**: Antifungal susceptibility profile of 24 *Pichia kudriavzevii* strains obtained from bloodstream infections in a neonatal intensive care unit.

|  | **Patient** | **Isolate ID** | **Median MIC values of antifungal drugs^a^ (** **mg/L)** | | | | | | | | |
| --- | --- | --- | --- | --- | --- | --- | --- | --- | --- | --- | --- |
|  |  |  | **FLU** | **ITC** | **VRC** | **ISA** | **POS** | **AMB** | **MFG** | **AFG** | **FC** |
| Cluster I | 1 | 1313/P/15 | 16 | 1 | 0.12 | 0.03 | 0.5 | 0.5 | 0.12 | 0.03 | 8 |
|  | 2 | 03/P/16 | 16 | 0.5 | 0.06 | 0.03 | 0.25 | 1 | 0.12 | 0.06 | 4 |
|  | 3 | 06/P/16 | 32 | 0.12 | 0.12 | 0.06 | 0.25 | 1 | 0.12 | 0.12 | 2 |
|  | 4 | 02/P/16 | 16 | 0.25 | 0.06 | 0.06 | 0.12 | 0.06 | 0.06 | 0.06 | 4 |
| Cluster IIa | 5 | 1596/P/18 | 32 | 0.5 | 0.06 | 0.06 | 0.25 | 0.5 | 0.12 | 0.12 | 8 |
|  | 6 | 1732/P/18 | 16 | 0.25 | 0.03 | 0.06 | 0.06 | 0.5 | 0.12 | 0.06 | 4 |
|  | 7 | 1734/P/18 | 32 | 0.06 | 0.12 | 0.03 | 0.25 | 0.12 | 0.12 | 0.12 | 4 |
|  | 8 | 404/P/19 | 16 | 0.5 | 0.06 | 0.03 | 0.25 | 0.5 | 0.25 | 0.5 | 8 |
|  | 9 | 577/P/19 | 16 | 0.12 | 0.12 | 0.06 | 0.25 | 0.25 | 0.12 | 0.12 | 4 |
|  | 10 | 581/P/19 | 32 | 0.5 | 0.06 | 0.06 | 0.25 | 1 | 0.12 | 0.06 | 4 |
|  | 11 | 601/P/19 | 16 | 0.06 | 0.12 | 0.03 | 0.25 | 0.06 | 0.06 | 0.06 | 4 |
|  | 12 | 677/P/19 | 16 | 0.25 | 0.25 | 0.25 | 0.25 | 1 | 0.12 | 0.06 | 16 |
|  | 13 | 723/P/19 | 32 | 0.5 | 0.25 | 0.25 | 0.25 | 1 | 0.12 | 0.06 | 16 |
| Cluster IIb | 14 | 177/P/20 | 16 | 0.5 | 0.06 | 0.06 | 0.25 | 1 | 0.25 | 0.5 | 8 |
|  | 15 | 205/P/20 | 16 | 0.5 | 0.03 | 0.06 | 0.25 | 0.25 | 0.12 | 0.5 | 8 |
|  | 16 | 218/P/20 | 8 | 0.5 | 0.06 | 0.06 | 0.25 | 0.5 | 0.12 | 0.5 | 8 |
|  | 17 | 248/P/20 | 8 | 0.25 | 0.03 | 0.03 | 0.12 | 0.5 | 0.25 | 0.5 | 8 |
|  | 18 | 251/P/20 | 16 | 0.25 | 0.06 | 0.03 | 0.25 | 0.25 | 0.25 | 0.5 | 8 |
|  | 19 | 255/P/20 | 8 | 0.5 | 0.06 | 0.06 | 0.25 | 0.5 | 0.25 | 0.5 | 8 |
|  | 20 | 261/P/20 | 16 | 2 | 0.12 | 0.12 | 0.5 | 1 | 0.25 | 0.25 | 8 |
|  | 21 | 263/P/20 | 8 | 0.5 | 0.06 | 0.03 | 0.25 | 0.5 | 0.25 | 0.5 | 8 |
|  | 22 | 267/P/20 | 16 | 0.5 | 0.03 | 0.03 | 0.25 | 0.5 | 0.25 | 0.5 | 8 |
|  | 23 | 272/P/20 | 16 | 0.25 | 0.06 | 0.06 | 0.25 | 0.12 | 0.25 | 0.5 | 8 |
|  | 24 | 240/P/20 | 16 | 0.25 | 0.03 | 0.03 | 0.12 | 0.5 | 0.12 | 0.5 | 8 |

^a^FLU, fluconazole ITC, itraconazole; VRC, voriconazole; ISA, isavuconazole; POS, posaconazole; AMB, amphotericin B; MFG, micafungin; AFG, anidulafungin; 5 FC, flucytosine;

^b^F, female;

^c^M, male
