# Supplementary material for: Genomic analyses reveal high diversity and rapid evolution of Pichia kudriavzevii within a neonatal intensive care unit in Delhi, India
Source: Antimicrob Agents Chemother. 2025 Jan 24;69(3):e01709-24. doi: 10.1128/aac.01709-24 (PMC11881565; doi:10.1128/aac.01709-24)
Supplement: Table S4 — In vitro susceptibility profile and point mutations in ERG11 gene of P. kudriavzevii strains obtained from patients admitted in the intensive care units of six hospitals. [file aac.01709-24-s0009.docx]

**Table S4:** *In vitro* susceptibility profile, and point mutations in *ERG11* gene of *Pichia kudriavzevii* strains obtained from patients admitted in the intensive care units of six hospitals.

| **S. No.** | **No. of Isolate** | **Specimen** | **Hospital** | **Drugs^a^ (MIC, mg/L)** | | | **Amino acid substitution in ERG11p** | **Synonymous mutation** |
| --- | --- | --- | --- | --- | --- | --- | --- | --- |
|  |  |  |  | ITC | FLU | POS |  |  |
| 1 | 1492/P/16 | Urine | Hospital A | 0.125 | 8 | 0.015 |  | C1470T |
| 2 | 1615/P/16 | Urine | Hospital A | 0.25 | 8 | 0.5 |  |  |
| 3 | 1619/P/16 | Urine | Hospital A | 0.06 | 4 | 0.25 |  |  |
| 4 | 1015/17 | Sputum | Hospital B | 0.125 | 8 | 0.015 |  | C1470T |
| 5 | 930/p/17 | Urine | Hospital C | 0.06 | 8 | 0.5 |  |  |
| 6 | 1285/P/17 | Blood | Hospital C | 0.125 | 8 | 0.5 | A15V | T642C |
| 7 | 640/p/18 | Blood | Hospital D | 0.03 | 8 | 0.125 | A15V |  |
| 8 | 604/p/18 | Blood | Hospital D | 0.03 | 8 | 0.25 | A15V | T642C, C1389T |
| 9 | 1394/P/18 | Blood | Hospital E | 0.06 | 8 | 0.125 | A15V | T642C |
| 10 | 1438/P/18 | Blood | Hospital E | 0.03 | 8 | 0.125 | A15V | T642C, A756T, C1389T |
| 11 | 1480/P/18 | Blood | Hospital E | 0.125 | 8 | 0.125 |  | T642C, C1027T, C1389T |
| 12 | 1443/P/18 | Blood | Hospital E | 0.5 | 32 | 0.25 |  | T642C, C1027T, C1389T |
| 13 | 1733/P/18 | Blood | Hospital E | 0.25 | 32 | 0.06 | A15V |  |
| 14 | 1390/P/18 | Blood | Hospital E | 0.06 | 32 | 0.5 | A15V | C1389T |
| 15 | 1395/P/18 | Blood | Hospital E | 0.125 | 32 | 0.5 | A15V | C1389T |
| 16 | 1397/P/18 | Blood | Hospital E | 0.125 | 32 | 0.5 | A15V |  |
| 17 | 1380/P/18 | Blood | Hospital F | 0.125 | 32 | 0.06 | A15V |  |
| 18 | 789/P/19 | Blood | Hospital E | 0.06 | 32 | 0.125 | A15V | C1470T |

^a^ITC, itraconazole; FLU, fluconazole; POS, posaconazole
