# Supplementary material for: Genomic analyses reveal high diversity and rapid evolution of Pichia kudriavzevii within a neonatal intensive care unit in Delhi, India
Source: Antimicrob Agents Chemother. 2025 Jan 24;69(3):e01709-24. doi: 10.1128/aac.01709-24 (PMC11881565; doi:10.1128/aac.01709-24)
Supplement: Fig. S1 — Read depth plot for 28 P. kudriavzevii strains across region NC_042509.1:613002-642999. [file aac.01709-24-s0001.pdf]

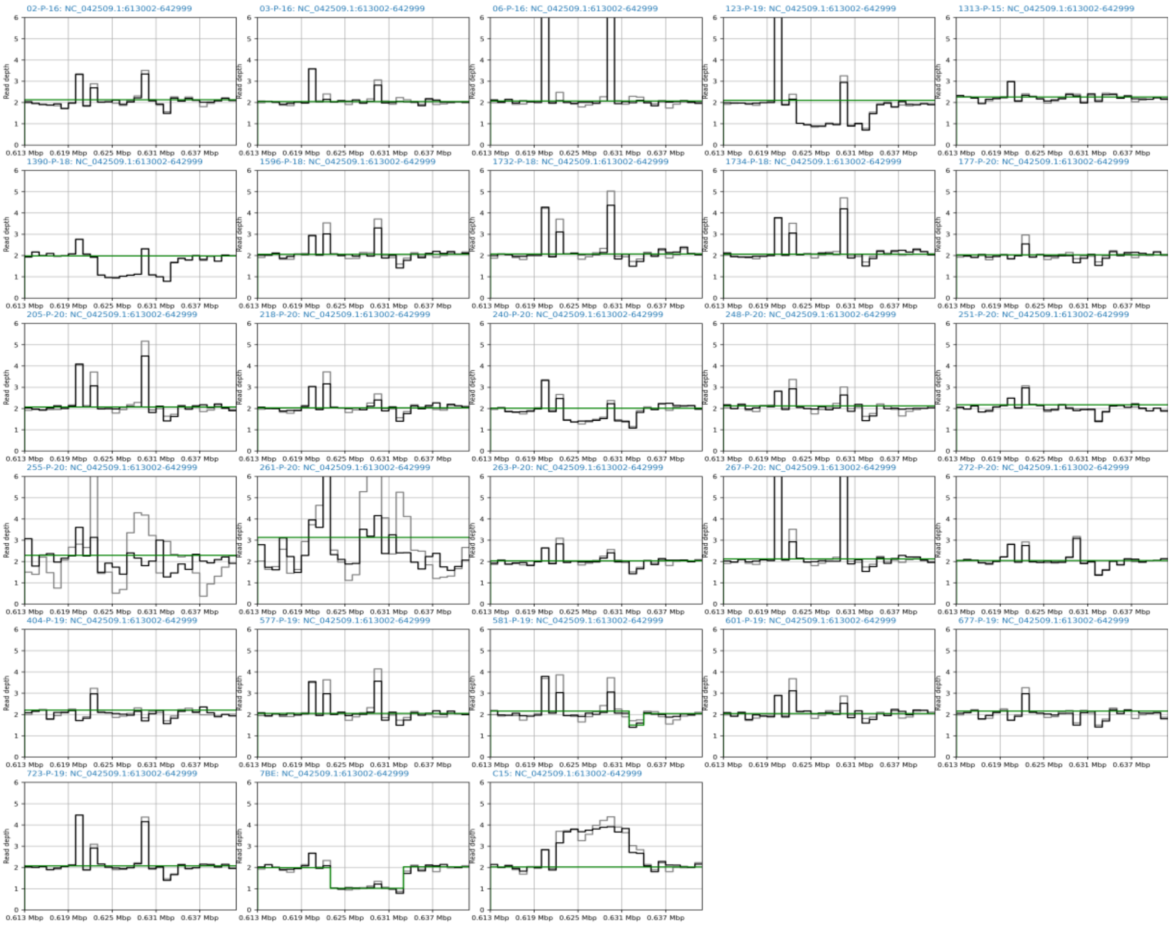

Figure S1: Read depth plot for 28 *P. kudriavzevii* strains across the region NC_042509.1:613002-642999
